# Restoration versus reconstruction: cellular mechanisms of skin, nerve and muscle regeneration compared

**DOI:** 10.1186/2050-490X-1-4

**Published:** 2013-10-01

**Authors:** Dario Coletti, Laura Teodori, Zhenlin Lin, Jean Francois Beranudin, Sergio Adamo

**Affiliations:** UPMC Univ Paris 06, UR4 Ageing, Stress, Inflammation, 75005 Paris, France; Department of Anatomical, Histological, Forensic & Orthopaedic Sciences, Section of Histology & Medical Embryology, 00161 Rome, Italy; Interuniversity Institute of Myology, Kragujevac, Italy; ENEA-Frascati, UTAPRAD-DIM, Diagnostics and Metrology Laboratory, 00044 Rome, Italy; Hôpital Tenon, Histology and Tumor Biology, UPMC Univ Paris 06, 75020 Paris, France

**Keywords:** Damage, Necrosis, Regeneration, Differentiation, Epithelial tissue, Nervous tissue, Skeletal muscle tissue, Skin scar, Stem cells, Extra cellular matrix

## Abstract

In tissues characterized by a high turnover or following acute injury, regeneration replaces damaged cells and is involved in adaptation to external cues, leading to homeostasis of many tissues during adult life. An understanding of the mechanics underlying tissue regeneration is highly relevant to regenerative medicine-based interventions. In order to investigate the existence a *leitmotif* of tissue regeneration, we compared the cellular aspects of regeneration of skin, nerve and skeletal muscle, three organs characterized by different types of anatomical and functional organization. Epidermis is a stratified squamous epithelium that migrates from the edge of the wound on the underlying dermis to rebuild lost tissue. Peripheral neurons are elongated cells whose neurites are organized in bundles, within an endoneurium of connective tissue; they either die upon injury or undergo remodeling and axon regrowth. Skeletal muscle is characterized by elongated syncytial cells, i.e. muscle fibers, that can temporarily survive in broken pieces; satellite cells residing along the fibers form new fibers, which ultimately fuse with the old ones as well as with each other to restore the previous organization. Satellite cell asymmetrical division grants a reservoir of undifferentiated cells, while other stem cell populations of muscle and non-muscle origin participate in muscle renewal. Following damage, all the tissues analyzed here go through three phases: inflammation, regeneration and maturation. Another common feature is the occurrence of cellular de-differentiation and/or differentiation events, including gene transcription, which are typical of embryonic development. Nonetheless, various strategies are used by different tissues to replace their lost parts. The epidermis regenerates *ex novo*, whereas neurons restore their missing parts; muscle fibers use a mixed strategy, based on the regrowth of missing parts through reconstruction by means of newborn fibers. The choice of either strategy is influenced by the anatomical, physical and chemical features of the cells as well as by the extracellular matrix typical of a given tissue, which points to the existence of differential, evolutionary-based mechanisms for specific tissue regeneration. The shared, ordered sequence of steps that characterize the regeneration processes examined suggests it may be possible to model this extremely important phenomenon to reproduce multicellular organisms.

## Review

### The importance of tissue regeneration in physiology and pathology

When talking of wound healing, a distinction is made by some authors between regeneration and repair. Regeneration is used to refer to the complete replacement of damaged tissue with new tissue not associated with scar tissue, while repair is used to refer to the re-establishment of tissue continuity [1]. Regeneration can be attained by two means: a) restoration, defined as “putting together what is broken”; b) reconstruction, defined as “replacing and rebuilding what is torn down” (according to the Merriam-Webster Dictionary). To grant homeostasis, most tissues undergo continuous or cyclic processes of regeneration. Which of the afore-mentioned strategies tissues adopt depends on the histological features discussed below.

Defects in wound repair constitute a severe health problem that frequently affects aged individuals, patients with diabetes or patients treated with immunosuppressants or chemotherapy [2]. An early hypothesis postulated that chronic irritation, previous injuries and consecutive repairs are a precondition for tumorigenesis [3]. This hypothesis has recently been reviewed and updated by Shafer and Werner, who referred to cancer as an overhealing wound [4]. Since the advent of regenerative medicine, tissue regeneration has attracted growing interest on account of its potential consequences on tissue engineering and *in situ* guided tissue regeneration [5].

This review presents and compares the cellular aspects of regeneration in skin, nerve and muscle, three organs characterized by differences not only in anatomical and functional organization, but also in the number and location of stem cell niches and populations, which ultimately result in varying regenerative potential. By discussing the common traits and the specific features of regeneration in three model tissues, we propose general models of regeneration and highlight various strategies adopted to cope with damage and repair in mammals. The mechanisms of cell differentiation underlying normal homeostasis of tissue characterized by a high turnover, due to short cell life or significant cell loss, do not fall within the scope of this review. We will focus, instead, on regeneration following acute injury.

### Common phases of tissue damage and regeneration

Regeneration *sensu lato* consists, in most tissues, of three phases: inflammation, repair and maturation. Following injury, cells are either quickly repaired or undergo necrosis, i.e. cell death characterized by rupture of the cell membrane and release of intracellular factors. The latter induce inflammation, which is required for the subsequent phase of regeneration. Examples of factors released by disrupted cells are: factor VIII, released by the endothelium [6]; Wnt, released by muscle fibers [7]; cell membrane-derived arachidonic acid metabolites, released by peripheral neurons [8]. Acute inflammation is characterized by the arrival of neutrophils and macrophages, which are responsible not only for the phagocytosis of dead cell debris but also for the production of the anti-inflammatory cytokines required for the down-regulation of the inflammatory response that prevents chronicization and further damage. The regulation of this shift in the inflammatory response has been described in many tissues, including skeletal muscle [9]. A clear example of this mechanism is the shift from the M1 to the M2 macrophage population [10], which is ultimately responsible for the passage from a necrotic environment to one favorable to stem cell homing and differentiation, which in turn results in tissue repair [11]. The latter is accomplished by resident and, occasionally, recruited stem cells, which proliferate and migrate to the site of damage during the inflammatory phase. Their proliferation is not only needed to provide a sufficient number of cells for differentiation and repair of extended damage, but also to reconstitute the tissue-specific stem cell pool. For this purpose, stem cell proliferation is characterized by asymmetric cell division [12]. Cell migration has recently been the object of exhaustive reviews [13–15]. Adult stem cells are extremely sensitive to the anatomy and the physicochemical nature of the environment, differentiating according to their specific niche, which in turn finely tunes the reconstitution of the tissue-specific stem cell pool. The fate of daughter stem cells may be determined by their orientation in relation to the surrounding cells, as exemplified by the fact that a planar versus apical-basal division of satellite cells in muscle determines the prevalence of symmetrical and asymmetrical divisions. Asymmetrical division is determined by asymmetrical (toward the muscle fiber side) cell expression of adhesion molecules such as M-CAD [16], which ultimately leads to marked expression of transcription factors such as Pax7; this in turn generates distinct daughter cell fates by asymmetrically segregating template DNA strands to the cell progeny [17]. A similar process appears to exist in epithelial and neural stem cells [18, 19].

The subpopulation of stem cells that undergo differentiation is directly responsible for tissue regeneration. Typically, differentiation is driven by master genes that progressively lead to the acquisition of the tissue-specific phenotype [20, 21]. Not surprisingly, the genes leading to stemness or differentiation are reciprocally antagonistic and inhibit each other.

Maturation, which is the last phase of regeneration, consists in the consolidation of a terminally differentiated phenotype. The tissue architecture does not change significantly in this phase, but the cells acquire a functionally mature phenotype. For instance, although sarcomerogenesis occurs upon differentiation, the original, embryonic isoforms of the contractile proteins expressed by newborn fibers are subsequently replaced by other isoforms that are typical of adult tissue [22].

Inflammation, differentiation and maturation differ from tissue to tissue in terms of the kinetics, mechanisms and final morphology of the newborn tissue. Details of the three model tissues described in this review are presented below.

### Skin regeneration

Following injury, inflammatory cell-derived proteinases degrade the blood clot, while the release of mitogens and chemoattractants by degranulating platelets stimulates migration and hyperproliferation of keratinocytes at the wound edge. Keratinocytes move between the blood clot and the underlying dermis as a monolayer sheet that subsequently undergoes multilayered stratification [13]. The shift in keratinocyte movement is regulated by progressive changes in the extra-cellular matrix (ECM) architecture and stiffness, as well as by autocrine-regulated cellular features, such as expression of cell adhesion molecules and cytoskeletal reorganization [14]. To ensure efficient migration, keratinocytes at the wound edge rearrange their actin cytoskeleton, extend lamellipodia and lose their cell–cell contacts, but maintain expression of integrin receptors to allow attachment to new substrates [23]; such events are reminiscent of the developmental process of epithelial–mesenchymal transition [24, 25] that also occur in malignancies [26]. The matrix, which works as a substratum for cell migration, arises from extravasated plasma fibronectin and *de novo* production of ECM proteins, such as vitronectin and thrombospondins, and soon also harbors fibroblasts and immune cells. The latter, in turn, stimulate keratinocyte proliferation and migration; the importance of populations of neutrophils, leukocytes and mast cells has been partially highlighted in transgenic animal models, but remains a matter of debate [27–29]. The new tissue that fills the wound, substituting the blood clot, is known as granulation tissue. Although it is readily vascularized through VEGF-mediated angiogenesis [30], a series of pro-angiogenic growth factors (including FGF2, HGF and granulocyte–macrophage colony stimulating factor) and negative regulators of angiogenesis (thrombospondin-1) are expressed in granulation tissue.

Following hyperproliferation and migration, keratinocytes differentiate, as recently reported by Simpson [31]. A subset of the fibroblasts that proliferate in the granulation tissue then differentiate into myofibroblasts, which are responsible for the deposition of additional matrix proteins and wound contraction. During the tissue remodeling phase, the initial collagen type III of the granulation tissue is gradually replaced by collagen type I, and the resultant larger collagen fibrils are abnormally arranged in parallel bundles. These processes result in the formation of a scar that contains dense connective tissue whose tensile strength and elasticity is lower than that of normal skin [32]. At the same time, myofibroblasts are responsible for wound closure through connective tissue contraction, entailing incremental shortening of the ECM material induced by the myofibroblasts [33, 34]. When re-epithelialization is complete, an important decrease in the number of cellular elements, and in particular of myofibroblasts, occurs as a result of apoptosis in granulation tissue [34]. These events are summarised in Table 1.

**Table 1:** **Kinetics of wound healing of the epidermis: main phases and players**

	Destruction	Repair	Remodeling
	Days following injury		
**When**	1 to 3	4 to 7	8 to 14
**Where**	clot	granulation tissue (GT)	epidermis late GT
**What**	clotting	migration new tissue formation	hyperproliferation remodeling
**Who**	mast cells ^(1)^	keratinocytes ^(4)^	fibroblasts ^(7)^
	macrophages ^(2)^	myofibroblasts ^(5)^	myofibroblasts ^(8)^
	neutrophils ^(3)^	endothelial cells ^(6)^	
**How**	histamine ^(1)^	proteases ^(4)^	EGF ^(7)^
	cytokines ^(2)^	SDF1, HGF ^(5)^	
	ROS ^(3)^	VEGF ^(6)^	

Following injury, regeneration of the skin can be schematically divided in three main phases. In human skin, wound healing is accomplished in weeks. In addition to the timeline (When), each row indicates the tissue involved (Where), the main output (What), the cell type involved most (Who) and some of the main molecular mediators (How) responsible for the various phases of wound healing. Epidermal growth factor (EGF); fibroblast growth factor (FGF); hepatocyte growth factor (HGF); stromal cell-derived factor 1 (SDF1); vascular endothelial growth factor (VEGF). Matching superscripts highlight the cells that produce the corresponding growth factors.

Stem cells are located in three different areas of the skin: hair follicle bulges, inter-follicular areas of the surface epidermis and sebaceous glands; although the relative contribution to skin wound healing of each of these stem cell populations is still poorly characterized, the involvement of different stem cell populations in cutaneous wound healing appears feasible, at least in animal models [35, 36].

### Nerve regeneration

Owing to the significant length of neurons, a nerve transection most often cuts the axon, generating two cell fragments: the cell fragment that is distal to the injury site undergoes Wallerian (anterograde) degeneration, which is needed to create a microenvironment that favours axonal regrowth; the proximal cell fragment, consisting of part of the axon and the cell body, undergoes morphological changes (chromatolysis) that mirror metabolic changes and prepare regeneration and axonal elongation. The connective tissue (endoneurium) and the Schwann cells that surround individual axons in a peripheral nerve in most cases survive focal injury, with important consequences on the nerve regeneration mechanisms (discussed below). Varying neuron survival rates are observed following nerve avulsion or transection in different body districts [37, 38]. Constant delivery of a labile, cell body-synthesized survival factor (e.g. NMNAT2) is required to avoid Wallerian degeneration [39]. Defects that prevent its delivery, including axonal injury [40], axonal transport impairment [41], cell death [42] and disruption of protein synthesis in the cell body, all trigger Wallerian axon degeneration [39]. The neuronal intrinsic mechanisms of axon regeneration most worthy of note are (a) axonal membrane sealing [43, 44], (b) formation of a retraction bulb (the retracting, proximal segment of a severed axon) and (c) sprouting of a growth cone, the growing counterpart of a retraction bulb [45].

a) Disruption of the membrane integrity following injury transiently opens the axonal plasma membrane and causes rapid entry of extracellular ions, which results in axon depolarization, an event that is essential for the closure of the lesion sites in the peripheral nervous system (PNS) [46, 47]. Na^+^ appears to define the resealing site, since the Na^+^ influx from the lesion site diffuses along the transected axon but returns to normal resting values thanks to the action of Na^+^-K^+^ ATPase, thereby establishing a spatial-temporal gradient of Na^+^ along the transected axon [46, 48]. In addition, an active Ca^2+^ influx through voltage-dependent calcium channels activates calpain and phospholipase A2 (PLA2), which mediate membrane resealing [47]. Extracellular cues, such as factors deriving from both neural cells and macrophages, stimulate nerve degeneration/regeneration (see Table 2) [49, 50]. In this regard, since nerve injury is sometimes associated with clot formation, it should be borne in mind that platelet microparticles, which promote neural stem cell differentiation, may play a role in nerve regeneration [51].

b) A prominent feature of a regeneration-incompetent retraction bulb is the disorganization of microtubules, which ultimately leads to dying-back away from the lesion site. Wallerian degeneration activates Schwann cells to produce growth factors and to clear the myelin debris [48] through intrinsic processes as well as by attracting scavenger macrophages [52]. Schwann cells stimulate axonal growth by producing growth and survival factors, thereby providing guidance for successful PNS regeneration (see Table 2). Accordingly, injured axons upregulate the expression of receptors for these growth factors. Intriguingly, a direct comparison study of the optic nerve, as a model of the central nervous system (CNS) and of the sciatic nerve injury, as a PNS model, showed that regulation of the ciliary neurotrophic factor (CNTF) and its axonal receptor in the CNS differs from that in the PNS, thus pointing to the existence of a mechanism underlying their different regenerative capacity [53]. Several myelin-associated factors are present in the PNS following axonal injury, including myelin-associated glycoprotein (MAG) and oligodendrocyte myelin glycoprotein (OMgp) [54]: as they represent an inhibitory signal for axonal regeneration, they have to be removed by Schwann cells and macrophages.

c) The formation of a regeneration-competent growth cone is Ca^2+^-dependent and requires activation of calpains, PLA2 and PKC [55]. Growth cone formation is also likely to depend on the interaction between the local cytoskeleton and the surrounding environment. Several molecular players, such as DLK-1 (dual leucine zipper kinase 1), that reorient the microtubules and permit the extension of regrowing axons [56, 57] have been identified. By analogy with development, neurotrophins such as NT-3, NGF and BDNF are thought to play a pivotal role in promoting axonal growth [58, 59]. Fine sprouts emerge from the proximal axonal end, elongating in the distal segment in association with the proliferated Schwann cells, which line up to form ordered columns called bands of Bungner, while the endoneurial tubes, which often remain intact, guide nerve reorganization [60]. At the molecular level, this event depends on the interaction of growth cones that express integrins with components in the extracellular matrix, such as laminin. Rapid down-regulation and re-expression of integrins and associated ligands during nerve degeneration and regeneration have been correlated with successful regeneration of peripheral nerves [61, 62]. Since integrin directly interacts with MAG and mediates MAG-dependent repulsive growth, it has been suggested that myelin-mediated inhibition and laminin-mediated stimulation may compete with one another and converge on the integrin signaling to regulate timely axonal degeneration or regeneration [48]. Once it has reached its target, the growth cone switches to differentiation into a presynaptic terminal [45].

**Table 2:** **Kinetics of wound healing of the nerve: main phases and players**

	Destruction	Repair	Remodeling
	Days following injury		
**When**	2 to 3 (prolonged up to 7-14)	4 to weeks	8 to weeks
**Where**	cell body	proximal axon segment	distal axon segment
	injured axon terminal		
	distal axonal segment		
**What**	chromatolysis	growth cone sprouting and elongation	nerve remodeling (supernumerary axonal sprout degeneration) reconnection with target (muscle re-innervation)
	Wallerian degeneration (myelin clearance)		
**Who**	cell body ^(1)^	Schwann cells ^(4) (5)^	pericytes ^(7)^
	B cells, macrophages ^(2)^	axons ^(6)^	Schwann cells ^(8)^
	Schwann cells ^(3)^		muscle fibers ^(9) (10)^
**How**	hypertrophy, protein synthesis^(1)^	NGF ^(4)^	CNTF ^(7)^ ^(8)^
	immune response ^(2)^	BDNF ^(5)^	IGF-1^(9)^
	MCP-1, LIF ^(3)^	NT-3 and−4/5 ^(6)^	FGF^(10)^

Following injury, regeneration of the nerve can be schematically divided in three main phases. Complete nerve regeneration in humans depends to a large extent on the length of the gap to be filled and may take many weeks. In addition to the timeline (When), each row indicates the tissue involved (Where), the main output (What), the cell type involved most (Who) and some of the main molecular mediators (How) responsible for the various phases of wound healing. Brain-derived neurotrophic factor (BDNF); ciliary neurotrophic factor (CNTF); fibroblast growth factor (FGF); insulin-like growth factor-1 (IGF-1); leukemia inhibitory factor (LIF); monocyte chemoattractant protein-1 (MCP-1); nerve growth factor (NGF); neurotrophin-3 (NT-3) and neurotrophin-4/5 (NT-4/5). Matching superscripts highlight the cells that produce the corresponding growth factors.

Although the involvement of autologous neural stem cells (NSC) in nerve wound repair is still unclear, adult brain-derived NCS grafting has been proposed as a potential approach for nerve repair [63]. Despite apparently being paradoxical since CNS regeneration does not seem to occur [64], NSC do possess self-renewal ability and differentiate into both mature neurons and gliocytes [19, 65].

### Skeletal muscle regeneration

**Figure 1:**
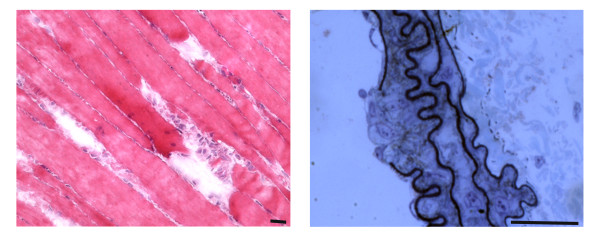
**Examples of focal injuries.** (LEFT) Hematoxilin-and eosin-stained murine skeletal muscle, longitudinally sectioned to show the gaps in three adjacent fibers. The injury likely occurred following an intense exercise session (wheel running). Upon leakage of the broken sarcolemma, factors such as Wnt are released before a fast repair process known as patch repair occurs. In turn, Wnt factors trigger the activation of satellite cells and other resident interstitial cells with myogenic potential, which proliferate, migrate and fuse into small myotubes that ultimately fuse with the damaged fibers. (RIGHT) Toluidine blue-stained semithin section of a murine carotid showing damage, likely due to smooth muscle cell-restricted inactivation of the serum response factor gene. A rupture of the endothelial layer, as well as of the elastin matrix, with exposure of underlying cells is visible; release of intracellular factors (von Willebrand Factor) and exposure of undisclosed antigens (collagen) are essential for the subsequent phases of clot formation, remodeling and repair of the wall defect. Bar = 25 micron.

**Figure 2:**
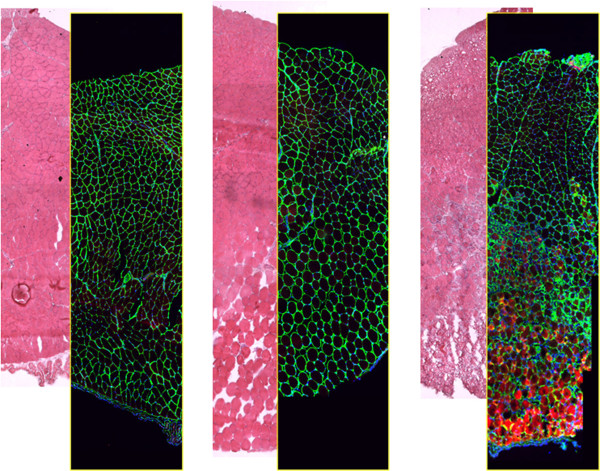
**Integrity of the extra cellular matrix following muscle injury.** Hematoxilin- and eosin-staining (H&E) and immunofluorescence localization of the membrane basement component laminin (green) on serial cross-sections of murine *Tibialis anterior* muscle (only a portion of the muscle is shown). Thirty minutes before fixation, the muscle was subjected to two types of physical injury: mechanical stress by crunching and tearing with forceps (LEFT) and freezing by applying a liquid nitrogen-cooled steel forceps to the surface (facing down in the picture) for 10 seconds (CENTER). Apart for the edema and fiber swelling visible in the images on the right, no major alterations of the basement membrane are seen following focal injury. In mice injected with Evans Blue Dye (EBD, RIGHT), injury muscle fiber necrosis (red) is apparent 8 h after freezing thanks to accumulation of EBD in the interior part of the damaged fibers. The muscle fibers die and are either renewed or replaced within the intact scaffold represented by the membrane basement, which wraps each fiber.

As an exhaustive monograph on skeletal muscle regeneration has recently been published [66], we will focus on certain unique features of skeletal muscle fibers that are particularly relevant to regeneration, such as their large size, elongated shape and syncytial nature. Since myofibers can be several millimeters in length, muscle injury and consequent skeletal muscle fiber necrosis are usually segmental (Figure 1, Table 3). Regeneration must be distinguished from various types of muscle fiber repair following different forms of muscle fiber damage that do not induce necrosis, with one example of the latter being patch repair, which restores sarcolemmal integrity by membrane resealing [67, 68]. Even when fiber necrosis (cell death) does occur, the overall extracellular matrix architecture and chemical composition are often preserved (Figure 2). However, while the basement membrane persists as a scaffold, molecules such as collagen IV start disappearing from as early as day 1 [69]. The degradation of these ECM components may be chemotactic in a wide range of cells, including myoblasts. Proteolysis by metalloproteinases mainly contributes to the modulation of the cell surface and the extracellular matrix [70, 71]. Cell surface-associated heparan sulphate proteoglycans, such as syndecans, play a major role in myogenesis *in vivo*: they are abundant on the surface of myofibers and myogenic cells, and they bind to growth factors relevant to myogenesis.

**Table 3:** **Kinetics of wound healing of the muscle: main phases and players**

	Destruction	Repair	Remodeling
	Days following injury		
**When**	1 to 5	3 to 7	8 to weeks
**Where**	hematoma (between fiber stumps)	necrotic segment	newborn fibers regenerated segment
**What**	phagocytosis of necrotized tissue inflammation Satellite cell activation	myofiber formation (fusion of muscle precursor cells)	myofiber growth
		gap refilling (myotube fusion with each other and surviving fibers)	myofiber function (fiber type specif.) scar reorganization
**Who**	muscle fibers ^(1)^ ^(2)^	muscle fibers ^(4) (5)^	fiber cytoskeleton ^(7)^
	muscle fibers ^(3)^		nerve sprouts ^(8)^
	connective tissue ^(4)^		intervening scar ^(9)^
	macrophages ^(4)^		
	neutrophils ^(5)^		
**How**	HGF ^(1)^	IGF-1 and−2 ^(4)^	myofibril remodeling ^(7)^
	FGF-1-2-4 and-6 ^(2)^	IL-4 ^(5)^	nerve activity (frequency)^(8)^
	MSTN/GDF8 ^(3) (4)^		scar retraction ^(9)^
	LIF ^(3) (4)^		
	TNF ^(5)^		

Following injury, regeneration of the muscle can be schematically divided in three main phases. Complete muscle regeneration in humans may take several weeks. In addition to the timeline (When), each row indicates the tissue involved (Where), the main output (What), the cell type involved most (Who) and some of the main molecular mediators (How) responsible for the various phases of wound healing. Matching superscripts highlight the cells that produce the corresponding growth factors or the cellular structures responsible for a given function. Fibroblast growth factor (FGF); hepatocyte growth factor (HGF); insulin-like growth factor (IGF); interleukin-4 (IL-4); leukemia inhibitory factor (LIF); myostatin (MSTN), also known as growth differentiation factor 8 (GDF-8); tumor necrosis factor (TNF).

**Figure 3:**
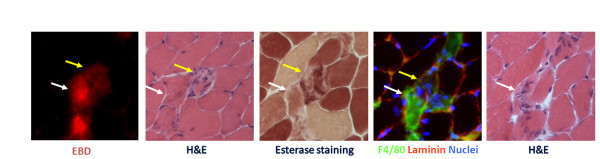
**Macrophages infiltrate necrotic muscle fibers.** Serial section of murine skeletal muscle in an area of necrosis; corresponding fibers are highlighted by matching color arrows. Evans Blue Dye (EBD) highlights muscle fibers whose plasma membrane is leaking owing to damage; hematoxylin and eosin staining (H&E), showing cellular infiltration in EBD + fibers; histochemistry for esterase staining highlights esterase-enriched macrophages; immunofluorescence analysis for activated macrophages expressing F4/80 (green) confirms the invasion of the muscle fibers by macropohages: laminin (red) and nuclei (blue) are also shown.

Fiber necrosis is the most common form of muscle damage. Though believed to be implicated in human muscle diseases, apoptosis is not an established means of muscle fiber extinction [72–74]. The surviving segments of the myofiber, on either side of the necrotic area, are readily sealed by a specific structure called the contraction band, a condensation of cytoskeletal material that acts as a system of “fire doors”. Within hours of injury, the propagation of necrosis is reduced to a local process [75]. The ruptured myofibers contract and the gap between stumps is filled by a hematoma [75]. Macrophage-mediated phagocytosis of the necrotic fiber segments is an essential prerequisite for optimal regeneration (Figure 3). The interposed scar gradually decreases in size, thereby bringing the stumps closer together until the myofibers become interlaced, though most likely not yet reunited. At the same time, inflammation activates the satellite cells [76], which consist of small, spindle-shaped, dormant mononuclear cells located between the basal lamina and the sarcolemma. Once they have been activated and are proliferating, these cells are referred to as myoblasts and express muscle regulatory transcription factors (MRF) that regulate cell cycle exit and differentiation. Other populations of resident [77] and circulating stem cells with myogenic potential may be involved in muscle regeneration in adulthood [78–80].

**Figure 4:**
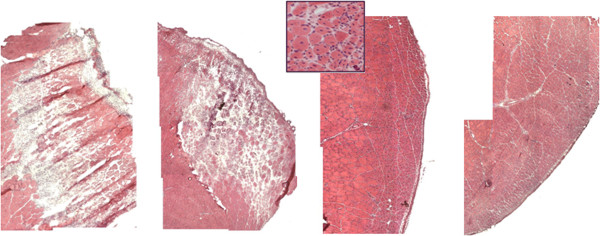
**Kinetics of skeletal muscle regeneration following focal injury in mouse.** Hematoxilin and eosin staining (H&E) of murine *Tibialis anterior* muscle (only a portion of the muscle is shown) subjected to freezing by applying a liquid nitrogen-cooled steel forceps to the surface (facing right in the picture) of the muscle for 10 seconds. The muscle was collected and analyzed 3, 6, 8 and 10 days following injury (from left to right). The inset shows a higher magnification image of regenerating fibers 8 days after injury: hallmarks of regenerating fibers include reduced fiber size and centrally located nuclei.

Following proliferation and migration from neighboring fibers, the myoblasts fuse with each other to form strips of multinucleated myotubes, a phenomenon observed during myogenesis *in vitro*[81] and during embryonic development *in vivo*[82, 83]. The nascent, newly formed bundles of myotubes, still adhering to the basal lamina, rapidly grow in diameter owing to generation of myofibrils organized in sarcomeres. As the girth of the myotubes increases, their sides come into contact with one another and they undergo lateral fusion as well as fusion to the surviving stump of the fiber, thereby generating newly-formed multinucleated regenerating muscle fibers. The latter are characterized by large, centrally-located myonuclei, prominent nucleoli and basophilic cytoplasm, indicating vigorous transcriptional and translational activity (Figure 4). Young, regenerating fibers are, on account of these distinguishing features, routinely used as markers of myopathies [84].

Regenerating myofiber behaves as if it were denervated until the regenerated segment with its newly formed endplate re-establishes normal nerve muscle contact, which in turns affects fiber physiology [85]. Worthy of note is the fact that nerve dependence of limb regeneration has been reported in non-mammal vertebrates [86]. Ultimately, the caliber of the regenerating fibers attains that of the fiber before necrosis (fiber maturation) and the regenerative markers disappear.

### Factors involved in the choice between restoration and reconstruction for tissue regeneration

Proteomic analysis and high throughput screening are providing a plethora of information on regenerating versus non-regenerating tissue, and will certainly help to clarify the events that might lead to the selection of one regenerative strategy rather than another, including cell signaling, transcription, metabolism and energetics, and cell protection, survival and cycle [87, 88]. All of these cell-intrinsic factors are likely to prove very important. In addition, the composition of both the preserved ECM and the matrix metalloproteinase-derived biodegradation products not only differ from tissue to tissue but also perform different biological activities (favoring cell adhesion and survival rather than cell migration and proliferation), ultimately affecting the capacity of the cells deputed to regeneration to perform one task (restoration) or the other (complete reconstruction) [89]. Wound oxygenation may, depending on the state of preservation of the capillary network, be a key factor in the healing process; mild hypoxia stimulates angiogenesis, collagen formation and cell survival, while extreme hypoxia delays healing [90].

One major issue concerns the overall amount of energy spared by choosing restoration rather than reconstruction to replace damaged tissues. Nutritional needs in metabolic disorders associated with severe wounds, such as cachexia [82], are critical during rehabilitation and recovery [91], highlighting the importance of energy balance in regeneration. Upon injury, stem cells exit quiescence to divide and differentiate; these opposing actions require distinct metabolic programs to meet the changing energy demands [92]. It is self-evident that a nerve undergoes an efficient, energy-sparing process by recycling its surviving cells to restore continuity (thus preserving its upstream connections), whereas skin tissue is characterized by large territories that are re-colonized through massive proliferation and migration of huge numbers of new cells. However, we believe that the increasing complexity of organ architecture may be the most important factor involved in the choice between restoration and reconstruction. The highly hierarchical muscle or nerve organization requires a process of regeneration *ex novo* that is so orchestrated and gradual that it would only be possible during embryogenesis or if mammals had conserved the epimorphic regeneration (in which a blastema of undifferentiated cells is formed) that is typical of other organisms and is capable of complete organ regeneration throughout adult life. The evolutionary and anatomical aspects of regeneration have recently been discussed; in this regard, it has been noted that “the complexity of mammalian tissues/organs seems to go in parallel with high heterogeneity in the distribution/features of stem cell compartments”, which are markedly different in perennial and labile tissues [93]. This may be considered another key factor involved in the choice of tissue-specific regeneration strategies.

## Conclusions

A damaged tissue whose cells cannot be repaired by intrinsic cellular mechanisms, such as membrane resealing, DNA repair, cell cycle arrest and cytoskeleton reorganization, undergoes three phases of tissue regeneration *sensu lato*: 1) a destruction phase, characterized by the rupture and ensuing necrosis of the cells, the formation of a hematoma and the inflammatory cell reaction (with phagocytosis of the necrotized tissue); 2) a repair phase, consisting in the restoration or the *ex novo* reconstruction of the original cell type, either with or without the production of a connective tissue scar (concomitantly with capillary ingrowth within the injured area); 3) a maturation phase, characterized by tissue remodeling, inducing a shift toward the definitive pattern of gene expression, which ultimately leads to the rescue of the original physiological function (thanks also to the re-establishment of a full interaction between regenerating tissue and its surroundings) [75]. This classical scheme of tissue degeneration and regeneration applies very clearly to the tissues reviewed here, even though their embryonic origin is not the same and they display markedly different anatomical and cellular features. However, strategies used by a wide range of tissues to replace their lost parts vary, probably as result of evolutionary-based mechanisms for specific tissue regeneration. While the epidermis regenerates *ex novo*, neurons restore their missing parts; muscle fibers instead use a mixed strategy, based on the reconstruction of missing parts and on the generation of new fibers. These differential strategies are represented by the two terms used in the title to refer to different forms of regeneration: restoration, the attempt to re-establish the *status quo ante*, and reconstruction, a more radical response, characterized by *ex novo* cell colonization and tissue formation. The choice of either strategy is deeply influenced by the anatomy and the distribution/features of stem cell niches typical of a given organ. In addition, the energetic costs for either regenerative strategy are also likely to play an important role. The abstraction of divergences and analogies between different types of tissue regeneration might pave the way for the mathematical modeling of this process, thereby making a major contribution to both pathology and regenerative medicine.

## Abbreviations

BDNF: Brain-derived neurotrophic factor
CNS: Central nervous system
CNTF: Ciliary neurotrophic factor
DLK-1: Dual leucine zipper kinase 1
ECM: Extra-cellular matrix
EGF: Epidermal growth factor
FGF: Fibroblast growth factor
GDF-8: Growth differentiation factor 8
HGF: Hepatocyte growth factor
IGF: Insulin-like growth factor
IL-4: Interleukin-4
LIF: Leukemia inhibitory factor
MMP: Matrix metalloproteinases
MAG: Myelin-associated glycoprotein
MRF: Muscle regulatory transcription factors
MSTN: Myostatin
MCP-1: Monocyte chemoattractant protein-1
NGF: Nerve growth factor
NSC: Neural stem cells
NT: Neurotrophin
NMNAT2: Nicotinamide mononucleotide adenylyltransferase
OMgp: Oligodendrocyte myelin glycoprotein
PKC: Protein kinase C
PLA: Phospholipases A
PNS: Peripheral nervous system
ROS: Reactive oxygen species
SDF1: Stromal cell-derived factor 1
SRF: Serum response factor
VEGF: Vascular endothelial growth factor
TNF: Tumor necrosis factor.
